# Exposure to Asbestos and Increased Intrahepatic Cholangiocarcinoma Risk: Growing Evidences of a Putative Causal Link

**DOI:** 10.5334/aogh.3660

**Published:** 2022-06-13

**Authors:** Giovanni Brandi, Kurt Straif, Daniele Mandrioli, Stefania Curti, Stefano Mattioli, Simona Tavolari

**Affiliations:** 1 Division of Oncology, IRCCS, Azienda Ospedaliero-Universitaria of Bologna, IT; 2 Department of Experimental, Diagnostic and Specialty Medicine, University of Bologna, IT; 3 ISGlobal, Barcelona, Spain and Boston College, MA, US; 4 Cesare Maltoni Cancer Research Center, Ramazzini Institute, Bologna, IT; 5 Department of Medical and Surgical Sciences, University of Bologna, IT; 6 Department of Environmental and Preventive Sciences, University of Ferrara, IT

**Keywords:** intrahepatic cholangiocarcimona, asbestos, risk factors

## Abstract

To date the true global incidence of intrahepatic cholangiocarcinoma (iCCA) and the underlying risk factors remain to be fully defined, in particular, the role of occupational and environmental factors. Currently, the putative role of asbestos exposure as a risk factor for iCCA is gaining increased attention in the international scientific community and agencies.

In this commentary we review and integrate available epidemiological and mechanistic evidences that support a potential role of asbestos exposure in iCCA etiology.

## Introduction

Intrahepatic cholangiocarcinoma (iCCA) arises from the epithelial cells within the liver bile ducts and represents the second most common primary liver cancer after hepatocellular carcinoma (HCC). One of the major challenges in the field of iCCA is to define the true global incidence of this disease and the underlying risk factors. Although still considered a rare malignancy in Europe and USA, in the past decades age-standardized iCCA incidence appeared to steadily increase in most locations worldwide [1]. However, this trend needs to be interpreted with caution due to the lack of a separate code for iCCA, extrahepatic (eCCA) and perihilar (pCCA) cholangiocarcinomas in the historical versions of the International Classification of Diseases (ICD) coding system. It is undeniable, though, that the hospital charge of patients with a diagnosis of iCCA is increased in the last 15 years; this increase has been registered not only in USA, but also in other international referral centers for hepato-biliary cancers worldwide, including Italian hospitals [2,3]. An important issue that has been raised by some authors as a possible bias in the identification of the true iCCA global incidence is the misclassification of this disease as HCC or liver metastases. However, it is important to underline that in international referral centers for hepato-biliary cancers, the differential diagnosis among these hepatic lesions is quite easy. Indeed, apart from the morphology and the obvious search of the primary cancer (in case of suspect for liver metastases), a panel of specific antibodies for immunohistochemistry (IHC) analysis is routinely employed for diagnostic purpose and include: CK20(+), CK7(–/+), CDX2(+) for metastatic colorectal cancer (CRC); CK7(+), TTF1(–), p40(+) for squamous-cell lung cancer; CK7(+), TTF1(+), p40(–) for lung adenocarcinoma; synaptophisin(+), cromogranin(+), TTF1(+) for lung small-cell carcinoma (which is morphologically very different from iCCA anyway); HSA(+), arginase-1(+) and glutamine synthetase(+) for HCC; CK7(+), CK19(+) and CEAp(+) for iCCA. As the diagnostic reliability of this standardized procedures is recognized worldwide, the suspect of a misleading diagnosis may be ruled out with reasonable confidence. Overall, the data from the real world of clinical practice, along with the finding that about 21% of the diagnosed cancers of unknown primary sites (CUPs) are biliary tract cancers [4], seem to suggest that the global incidence of iCCA is likely underestimated than overestimated.

Currently certain pathological/genetic conditions (including primary sclerosing cholangitis, hepatolithiasis, bile duct cysts, Caroli’s disease, liver fluke infections and hemochromatosis type 1) have been recognized as risk factors for iCCA [1,5]. In East Asia (Thailand, Laos, Cambodia and Vietnam), where liver flukes are *endemic*, parasitic infections with *Clonorchis sinensis* and *Opisthorchis viverrini* represent the dominant risk factor for this disease [1]. A different scenario occurs in Western countries, where the associated risk factors still remain unknown in most of the diagnosed iCCA cases [1]. To date the role of occupational and environmental risk factors in iCCA development has been little investigated. An increased iCCA incidence has been reported among Japanese printing workers following chronic exposure to the volatile organic solvents 1,2-dichloropropane and dichloromethane [6,7]. Interestingly, whole-exome sequencing (WES) analysis of the tumor tissue of these workers has revealed a unique mutational profile and a mutation burden 30-fold higher to that observed in iCCAs of patients not exposed to these solvents [8]. However, as the global number of subjects exposed to 1,2-dichloropropane and dichloromethane is limited, other occupational and environmental risk factors need to be considered to explain the worldwide increase in iCCA incidence.

In this commentary we review current epidemiological and molecular evidence that support a potential link between asbestos exposure and increased iCCA risk.

## Asbestos and iCCA: Findings from epidemiological, molecular and histological studies

The possible association between asbestos exposure and biliary tract cancers was firstly observed some decades ago by Selikoff et al. who reported a significant increase (RR = 2.42, p < 0.01) of death for gall bladder/bile duct cancer among 17 800 asbestos insulation workers of USA and Canada from 1967 to 1986 [9]. This possible association has been investigated in subsequent cohort studies [10,11], even if the lack of distinction between iCCA and HCC makes the results of these studies difficult to interpret (Table 1). More compelling evidences have been provided from the first two case-control studies ever published on this topic [12,13]. The first study was carried out in Italy and included 69 iCCAs and 86 eCCAs patients matched up to four controls per case. An increased risk for iCCA in workers exposed to asbestos (OR = 4.81 95% CI 1.73–13.33) was observed, whereas limited evidence was found for eCCA (OR = 2.09 95% CI 0.83–5.27) [12]. The second case-control study was nested in the Nordic Occupational Cancer (NOCCA) cohort and included 1 458 iCCA and 3 972 eCCA cases from population-based cancer registries of Finland, Iceland, Norway and Sweden [13]. National decennial census data on occupations from 1960 to 1990 were linked to the NOCCA asbestos job-exposure matrix; notably it was observed an increasing risk of iCCA with cumulative exposure to asbestos, with an OR of 1.7 (95% CI 1.1 to 2.6) for subjects with a cumulative exposure of ≥15.0 f/mL × years compared to never exposed [13].

**Table 1:** Cohort and case-control studies investigating occupational asbestos exposure and iCCA risk.


TYPE OF STUDY	NO OF SUBJECTS	WORKERS’ CATEGORY	YEARS	ASBESTOS EXPOSURE ASSESSMENT	CANCER SITE	EFFECT MEASURES

Cohort[10]	• 4427 workers • 22135 matched controls	Shipbreaking workers	1975–1989	A panel of seven experts was asked to assess exposure subjectively	Liver and Intrahepatic bile ducts	HR_adj_ 1.6 (95% CI 1.08-2.36)

Cohort [11]	12578 workers	Asbestos-cement workers	1934–2006	Two expert industrial hygienists estimated asbestos exposure based on already collected data, for each plant and period	Liver and Intrahepatic bile ducts	SMR 0.99 (95% CI 0.81–1.20) (Males) SMR 0.87 (95% CI 0.42–1.60) (Females)

Case-control [12]	• 41 iCCA cases • 149 controls	All the different occupations of cases and controls were considered	2006–2010	Based on detailed entire job history and calendar periods, assessment of past asbestos exposure (Yes/No) was performed independently by two Occupational Physicians, unaware of case/control status	Intrahepatic bile ducts	Occupational exposed to asbestos *vs* not exposed OR_adj_ 4.81 (95% CI 1.73–13.33)

Case-control [13]	• 1458 iCCA cases • 6773 controls	All the different occupations of cases and controls were considered	1971–2005	The exposure to asbestos for each subject was estimated by applying the NOCCA job-exposure matrix (JEM) to the available occupational codes of cases and controls	Intrahepatic bile ducts	Cumulative exposure 1.0 (reference):0 f/mL × years OR _adj_ 1.1 (95% CI 0.9-1.3): 0.1–4.9 f/mL × years OR _adj_ 1.3 (95% CI 0.9-2.1): 5.0–9.9 f/mL × years OR _adj_ 1.6 (95% CI 1.0-2.5): 10.0–14.9 f/mL × years OR _adj_ 1.7 (95% CI 1.1-2.6): ≥15.0 f/mL × years


^a^ Estimates from logistic regression models conditioned on matching variables (year of birth, gender and country). Abbreviations: HR_adj_: adjusted hazard ratio; SMR: standardized mortality ratio; OR_adj_: adjusted Odds ratio; f/mL: fibers/mL; NOCCA: Nordic Occupational Cancer Study.

In order to hypothesize a role of asbestos exposure in iCCA development, the detection of fibers in the intrahepatic biliary tract is mandatory. Two recent exploratory studies reported the deposition of asbestos fibers in the bile/gallbladder of patients with benign diseases of the biliary tract and, more interestingly, in the liver of iCCA patients living in Casale Monferrato, an area of Italy at high level of environmental exposure to asbestos [14,15]. These findings are in line with previous studies showing that, beyond the respiratory tract, asbestos fibers may disseminate through other organs in the body, including the liver and the biliary tract [16,17]. Undoubtedly, the detection of fibers in the liver does not represent per se a sufficient condition to sustain a causal link between asbestos exposure and iCCA development, as they have been detected also in some cancers not related to asbestos exposure [16]. Nevertheless, this finding deserves further investigations to shed more light on the whole spectrum of extra-pulmonary cancers related to asbestos exposure. Indeed, the susceptibility to asbestos-induced carcinogenesis seems to vary among the different tissue types, making some organs at a higher cancer risk (or more prone to earlier cancer development) compared to other ones [9]. It is widely recognized that inflammatory response plays an important role in cancer onset and progression, and biliary tract diseases associated with chronic inflammation (primary sclerosing cholangitis, hepatolithiasis, choledochal cysts and liver fluke infections) have been established as risk factors for iCCA development [1]. Interestingly, thin and long asbestos fibers can induce a state of chronic inflammation in target tissues, due to their ability to persist for a very long time and to stimulate the prolonged release of pro-inflammatory cytokines by activated macrophages [18]. In this scenario, it is possible that asbestos fibers trapped in the smaller hepatic sinusoids may induce a state of chronic inflammation in the liver, similarly to what occurs in biliary tract diseases associated with chronic inflammation, leading to cell malignant transformation and cancer development.

From a molecular point of view, the knowledge of the mechanisms driving iCCA carcinogenesis is rapidly evolving due to the availability of high throughput analytical technologies such as next-generation sequencing. In lung cancer, genomic profiling identified a distinctive molecular signature in asbestos-exposed patients compared to not-exposed, including copy number aberrations in the 2p16, 9q33.1 and 19p13 loci and MRPL1, INPP4A, SDK and SEMA5B somatic mutations [19,20]. In malignant pleural mesothelioma, a classic model of asbestos-related cancer, BAP1 has been reported as the most frequently altered gene, with a frequency ranging from 23% to 57% of cases [21,22]. Similarly, a recent WES analysis on iCCA patients, categorized according to recognized risk factors for this disease and to the Italian National Mesothelioma Register (ReNaM) questionnaire for asbestos exposure, revealed a higher rate of BAP1 somatic mutations in asbestos-exposed patients compared to non-exposed (27% vs 5%, p-value = 0.0289) [23]. Furthermore, the first clinical case of a 47 years-old patient developing an iCCA in absence of risk factors, except for occupational exposure to low levels of asbestos for about 15 years, has been reported [24]. This patient, along with BAP1 loss of heterozygosity in tumor cells (a frequent genetic event in iCCA [25]), also carried a BAP1 germline mutation (c.255_255 + 6del). Interestingly, in humans, cells with BAP1 germline mutations have been shown to be more susceptible to asbestos carcinogenesis, because of their reduced ability to repair DNA damages and to trigger apoptosis following exposure to environmental carcinogens [26]. However, as BAP1 molecular alterations have been detected also in cancer patients occupationally not-exposed to asbestos [27,28], further studies are needed to better clarify the role of BAP1 gene in asbestos-induced carcinogenesis.

## Conclusions

Currently, about 125 million of people are still environmentally exposed to asbestos worldwide, even in countries that banned its use [29]. Recently, the possible role of asbestos exposure as a risk factor for iCCA is gaining increased attention in the international scientific community and agencies [30,31].

Overall, the body of evidences coming from epidemiological and mechanistic studies addresses to a putative causal role of asbestos in the genesis of iCCA, deserving further investigations in large observational studies with accurate asbestos exposure assessment.
